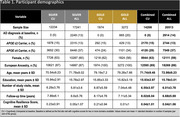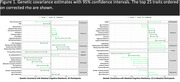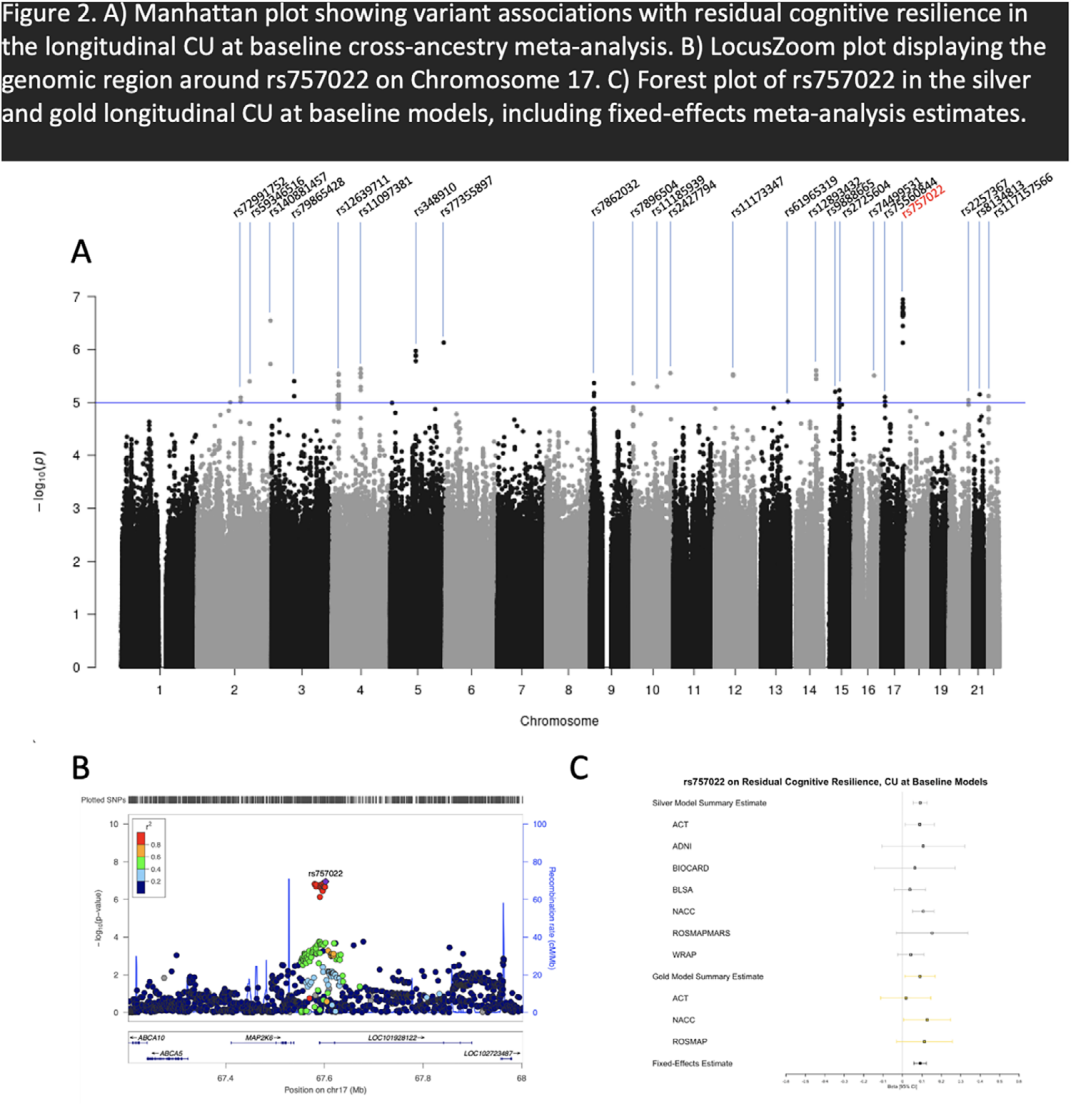# Leveraging a Novel Phenotype to Identify Complex Traits and Biological Pathways Associated with Resilience to Alzheimer’s Disease

**DOI:** 10.1002/alz.095693

**Published:** 2025-01-09

**Authors:** Jared Phillips, Logan C. Dumitrescu, Derek B. Archer, Alexandra N. Regelson, Shubhabrata Mukherjee, Michael L. Lee, Seo‐Eun Choi, Phoebe Scollard, Emily H. Trittschuh, Walter A. Kukull, Sarah A Biber, Jesse Mez, Emily R. Mahoney, Michelle Clifton, Julia B. Libby, Skylar Walters, William S. Bush, Corinne D. Engelman, Qiongshi Lu, David W. Fardo, Keith F. Widaman, Rachel F Buckley, Elizabeth Mormino, Elizabeth Sanders, Lindsay R. Clark, Katherine A. Gifford, Badri N. Vardarajan, Michael L. Cuccaro, Margaret A Pericak‐Vance, Lindsay A. Farrer, Li‐San Wang, Gerard D. Schellenberg, Jonathan L. Haines, Angela L. Jefferson, Sterling C. Johnson, Marilyn S. Albert, C Dirk Keene, Andrew J. Saykin, Shannon L. Risacher, Eric B Larson, Reisa A Sperling, Richard Mayeux, Alison M. Goate, Alan E. Renton, Edoardo Marcora, Brian Fulton‐Howard, Tulsi Patel, David A. Bennett, Julie A. Schneider, Lisa L. Barnes, Carlos Cruchaga, Jason J. Hassenstab, Michael E. Belloy, Shea J Andrews, Susan M. Resnick, Murat Bilgel, Yang An, Lori L Beason‐Held, Keenan A. Walker, Michael R Duggan, Brandon S Klinedinst, Paul K. Crane, Timothy J. Hohman, The Alzheimer’s Disease Sequencing Project (ADSP)

**Affiliations:** ^1^ Department of Pharmacology, Vanderbilt University, Nashville, TN USA; ^2^ Vanderbilt Memory & Alzheimer’s Center, Vanderbilt University Medical Center, Nashville, TN USA; ^3^ Vanderbilt Genetics Institute, Vanderbilt University Medical Center, Nashville, TN USA; ^4^ Department of Medicine, University of Washington, Seattle, WA USA; ^5^ Department of Psychiatry and Behavioral Sciences, University of Washington School of Medicine, Seattle, WA USA; ^6^ VA Puget Sound Health Care System, Seattle, WA USA; ^7^ National Alzheimer’s Coordinating Center, University of Washington, Seattle, WA USA; ^8^ Department of Neurology, Boston University Chobanian & Avedisian School of Medicine, Boston, MA USA; ^9^ Cleveland Institute for Computational Biology, Department of Population and Quantitative Health Sciences, Case Western Reserve University, Cleveland, OH USA; ^10^ University of Wisconsin‐Madison, School of Medicine and Public Health, Madison, WI USA; ^11^ Department of Statistics, University of Wisconsin‐Madison, Madison, WI USA; ^12^ Department of Biostatistics and Medical Informatics, University of Wisconsin‐Madison, Madison, WI USA; ^13^ Department of Biostatistics, College of Public Health, University of Kentucky, Lexington, KY USA; ^14^ Sanders‐Brown Center on Aging, University of Kentucky, Lexington, KY USA; ^15^ University of California at Riverside, Riverside, CA USA; ^16^ Center for Alzheimer’s Research and Treatment, Brigham and Women’s Hospital/Harvard Medical School, Boston, MA USA; ^17^ Melbourne School of Psychological Sciences, University of Melbourne, Melbourne, VIC Australia; ^18^ Department of Neurology, Massachusetts General Hospital, Harvard Medical School, Boston, MA USA; ^19^ Department of Neurology and Neurological Sciences, Stanford University, Stanford, CA USA; ^20^ University of Washington, Seattle, WA USA; ^21^ Geriatric Research Education and Clinical Center, William S. Middleton Memorial Veterans Hospital, Madison, WI USA; ^22^ Department of Neurology, Columbia University Medical Center, New York, NY USA; ^23^ Taub Institute for Research on Alzheimer’s Disease and the Aging Brain, Columbia University Medical Center, New York, NY USA; ^24^ The Institute for Genomic Medicine, Columbia University Medical Center, New York, NY USA; ^25^ John P. Hussman Institute for Human Genomics, University of Miami Miller School of Medicine, Miami, FL USA; ^26^ Dr. John T. Macdonald Foundation Department of Human Genetics, University of Miami Miller School of Medicine, Miami, FL USA; ^27^ Department of Medicine (Biomedical Genetics), Boston University Chobanian & Avedisian School of Medicine, Boston, MA USA; ^28^ Department of Biostatistics, Boston University School of Public Health, Boston, MA USA; ^29^ Penn Neurodegeneration Genomics Center, Department of Pathology and Laboratory Medicine, University of Pennsylvania Perelman School of Medicine, Philadelphia, PA USA; ^30^ Johns Hopkins University School of Medicine, Baltimore, MD USA; ^31^ Department of Laboratory Medicine and Pathology, University of Washington, Seattle, WA USA; ^32^ Indiana Alzheimer’s Disease Research Center, Indianapolis, IN USA; ^33^ Ronald M. Loeb Center for Alzheimer’s Disease, Friedman Brain Institute, Icahn School of Medicine at Mount Sinai, New York, NY USA; ^34^ Department of Genetics and Genomic Sciences, Icahn School of Medicine at Mount Sinai, New York, NY USA; ^35^ Rush Alzheimer’s Disease Center, Rush University Medical Center, Chicago, IL USA; ^36^ Rush Alzheimer's Disease Center, Rush University Medical Center, Chicago, IL USA; ^37^ Department of Psychiatry, Washington University School of Medicine, St. Louis, MO USA; ^38^ Department of Neurology, Washington University School of Medicine, St. Louis, MO USA; ^39^ Department of Psychiatry and Behavioral Sciences, University of California ‐ San Francisco, San Francisco, CA USA; ^40^ Laboratory of Behavioral Neuroscience, National Institute on Aging, Intramural Research Program, Baltimore, MD USA; ^41^ National Institute on Aging, National Institutes of Health, Baltimore, MD USA

## Abstract

**Background:**

Previous models of resilience to Alzheimer’s Disease (AD) have relied on cross‐sectional designs and inclusion of measures of neuropathology. Here, we present a novel modeling approach incorporating longitudinal data and the use of *APOE* and higher order interaction terms to approximate neuropathological resilience, vastly increasing participant diversity and statistical power. We validate this approach and report novel genetic associations with neuropathological resilience.

**Method:**

Leveraging 20,513 participants (Table 1), we explored the genetics of resilience metrics using mixed‐effects regressions across ancestries. We conducted GWAS on four resilience metrics: two in a “silver” model, which benefit from a larger sample size and did not include neuropathology at autopsy, and two in a “gold” model, which incorporate neuropathology at autopsy. These models were built in all diagnoses and separately in cognitively unimpaired (CU) at baseline participants. Silver‐gold meta‐analyses were conducted to maximize statistical power. Models adjusted for age, sex, *APOE* ε4 allele count, presence of the *APOE* ε2 allele and all covariate interactions with interval (years from baseline). Residual cognitive resilience was quantified from residuals in three cognitive domains (memory, executive function, and language). Post‐GWAS analyses included gene and pathway tests using MAGMA and genetic correlation with 65 traits using GNOVA.

**Result:**

Silver and gold resilience metrics were highly correlated (0.77‐0.88) and showed positive genetic covariance (0.01‐0.13). We identified negative correlations of resilience with multiple sclerosis and neuropsychiatric traits, including Tourette syndrome and schizophrenia (all P_FDR_<2.6×10^−2^; Figure 1). Furthermore, we observed three significant pathway associations with resilience: metabolism of amino acids and derivatives in the silver all participants meta‐analysis, negative regulation of transforming growth factor beta production in the silver CU at baseline meta‐analysis, and the SARS pathway in the gold CU at baseline meta‐analysis (all P_FDR_<5.0×10^−2^). Finally, we identified a locus on chromosome 17 associated with resilience that approached genome‐wide significance (top SNP: rs757022, MAF = 0.18, b = 0.08, P = 1.1×10^−7^; Figure 2).

**Conclusion:**

Using novel, longitudinal modeling approaches, we identified multiple biological pathways and complex traits associated with resilience to AD as well as genetic loci approaching genome‐wide significance. Our findings indicate that silver standard modeling approaches can complement current gold standard methods to accelerate genetic discovery.